# Whole genome sequencing reveals epistasis effects within RET for Hirschsprung disease

**DOI:** 10.1038/s41598-022-24077-w

**Published:** 2022-11-28

**Authors:** Yanbing Wang, Timothy Shin Heng Mak, Saloni Dattani, Maria-Merce Garcia-Barcelo, Alexander Xi Fu, Kevin Y. Yip, Elly Sau-Wai Ngan, Paul Kwang-Hang Tam, Clara Sze-Man Tang, Pak Chung Sham

**Affiliations:** 1 Department of Surgery, The University of Hong Kong, Hong Kong SAR, China; 2 Fano Labs, Hong Kong SAR, China; 3 Department of Psychiatry, The University of Hong Kong, Hong Kong SAR, China; 4 Social, Genetic and Developmental Psychiatry Centre, Institute of Psychiatry, Psychology and Neuroscience, King’s College London, London, UK; 5 Department of Computer Science and Engineering, The Chinese University of Hong Kong, Hong Kong SAR, China; 6 Sanford Burnham Prebys Medical Discovery Institute, La Jolla, CA 92037 USA; 7 Dr Li Dak-Sum Research Center, The University of Hong Kong-Karolinska Institute Collaboration in Regenerative Medicine, Hong Kong SAR, China; 8 Faculty of Medicine, Macau University of Science and Technology, Macao, China; 9 The State Key Laboratory of Brain and Cognitive Sciences, The University of Hong Kong, Hong Kong SAR, China

**Keywords:** Genetics, Genetic association study, Genetic interaction

## Abstract

Common variants in *RET* and *NRG1* have been associated with Hirschsprung disease (HSCR), a congenital disorder characterised by incomplete innervation of distal gut, in East Asian (EA) populations. However, the allelic effects so far identified do not fully explain its heritability, suggesting the presence of epistasis, where effect of one genetic variant differs depending on other (modifier) variants. Few instances of epistasis have been documented in complex diseases due to modelling complexity and data challenges. We proposed four epistasis models to comprehensively capture epistasis for HSCR between and within *RET* and *NRG1* loci using whole genome sequencing (WGS) data in EA samples. 65 variants within the Topologically Associating Domain (TAD) of *RET* demonstrated significant epistasis with the lead enhancer variant (*RET*+*3*; rs2435357). These epistatic variants formed two linkage disequilibrium (LD) clusters represented by rs2506026 and rs2506028 that differed in minor allele frequency and the best-supported epistatic model. Intriguingly, rs2506028 is in high LD with one cis-regulatory variant (rs2506030) highlighted previously, suggesting that detected epistasis might be mediated through synergistic effects on transcription regulation of *RET.* Our findings demonstrated the advantages of WGS data for detecting epistasis, and support the presence of interactive effects of regulatory variants in *RET* for HSCR.

## Introduction

Hirschsprung disease (HSCR), also known as colonic aganglionosis, is a congenital disorder where defects in enteric neural crest cell migration, differentiation or proliferations result in incomplete innervation of the distal gut. It exhibits features consistent with an appreciable degree of epistasis, which refers to the situation where the phenotypic effect of one genetic variant differs depending on the presence or absence of another genetic variant. Family studies estimate an overall heritability of HSCR close to 80%^1^, while the additive effects of identified common genetic variations explain only 34% of the phenotypic variance in Asian GWAS^2^. Isolated HSCR shows complex inheritance^3^, but the risk of HSCR is largely influenced by a small number of genes with moderately large effect. The *RET* gene is the major contributor of genetic predisposition to the disease, as almost all patients carry at least one *RET* deficiency allele^4^, and rare exonic mutations in *RET* explain up to 50% of familial cases of HSCR and up to 20% of isolated cases^5^. Furthermore, the effects of genetic variants on HSCR risk are multiplicative, with the number of implicated alleles being associated with an exponential increase in risk of developing the disease^6^.

The most prominent HSCR-associated gene, *RET*, is a tyrosine kinase receptor that is important for cell signalling and the development of the enteric nervous system. Epistasis has been detected within *RET* and between risk variants in *RET* and other genes, including *NRG1* and *EDNRB*^7^. Chatterjee et al*.*^4^ demonstrated a biological interaction in *RET*, in which a combination of *cis*-regulatory HSCR-associated variants (*RET*+*3* (rs2435357), *RET-5.5* (rs7069590), and *RET-7* (rs2506030)) synergistically reduced *RET* expression through both positive and negative feedback. In addition to *RET*, common variants in *NRG1* and *SEMA3C/D* have been consistently linked to HSCR through genome-wide association studies (GWAS), although evidence for *SEMA3C/D* has been restricted to European populations and evidence for *NRG1* is stronger in Asian populations^1,8,9^. Garcia-Barcelo et al.^10^ found a significant interaction between rs2435357 and rs7835688 from *NRG1*—the risk allele C in rs7835688 increased the odds ratio of rs2435357 2.3 times to almost 20. Such genetic interplay was later confirmed functionally^11^ and in an Indonesian cohort^12^. Likewise, the frequency of the rs2435357 risk variant (T) in *RET* was higher in patients with the rs12707682 risk allele in *SEMA3C/D* than in those without, which is in line with the synergistic effects on gut innervation observed in co-knockdown of *sema3c/d* and *ret* in the zebrafish model^8^.

Epistatic effects have been argued to be ubiquitous in nature^13^. In principle, epistasis can be identified by testing for interaction terms between genetic variants in regression models^14^. However, the detection of epistasis in genome-wide association studies (GWAS) involves testing a huge number of combinations of genetic variants, and therefore suffers from high computational burden and low statistical power after multiple testing adjustment. Furthermore, despite the likely biological ubiquity of epistasis, both theory and empirical data suggest that the total genetic variance of polygenic traits is likely to be largely explained by additive effects, with non-additive effects (dominance and epistasis) making a much smaller contribution^15–18^. There are some situations in which the amount of genetic variance contributed by epistasis can be larger than usual, and the characterisation of epistasis can substantially improve the accuracy of individual risk prediction. Epistasis can be more important when most of the genetic variance arises from a small number of loci with large individual effects, when there is high allele heterozygosity^16,18^, when the genotypic values have a non-linear relationship with the phenotype (e.g. as in a multiplicative model)^19^, or where interacting alleles are under strong linkage disequilibrium (LD)^20,21^. Moreover, even when the trait variance contributed by epistasis is small, its identification and quantification can still increase the precision and accuracy of individual risk estimation^20^. However, the detection of epistasis is challenging in GWAS, which relies largely on indirect association through LD. First, the reduction in statistical power due to incomplete LD between the causal and genotyped single nucleotide polymorphisms (SNPs) is greater for epistasis than for main effects. Secondly, the observed interaction effects between two genotyped SNPs can result from a hidden causal SNP that is tagged by a specific haplotype formed by the two SNPs, rather than true epistasis^22,23^. In this scenario, the apparent interaction between two SNPs would disappear if one could control for the untyped causal SNP. These issues can be resolved if we use whole-genome sequencing (WGS) to fully capture all SNPs in the genome.

In this study, we used the high-coverage WGS data on 443 HSCR cases and 493 controls to examine evidence of epistatic effects^24^; the use of WGS rather than GWAS data overcomes the problems of indirect association and hidden SNPs mentioned above. In order to reduce multiple testing and minimize false positive findings, we restricted the analysis to SNPs and short indels within the Topologically Associating Domains (TADs) encompassing *RET* and *NRG1*, since *cis* regulator elements for gene expression are likely to involve long-range physical interactions between chromosomal segments, and such interactions occur most frequently between chromosomal segments within the same TAD^25^. Tests for epistasis were performed between the most strongly associated variant (or lead variant) for each gene (rs2435357 in *RET* and rs7005606 in *NRG1*) and each of the other variants within the respective TADs. To explore the full range of possible patterns of interaction between the risk alleles, we considered four epistasis models: (1) a phase-independent model with interaction between both alleles present at one SNP and both alleles present at the other SNP, regardless of parental origin, (2) a *cis* model with interaction only between alleles inherited from the same parent (and are therefore on the same chromosome if the 2 SNPs are syntenic), (3) a *trans* model with interaction between alleles inherited from different parents, and (4) a *cis* and *trans* (C&T) model, where interaction between alleles inherited from the same parent and interaction between alleles inherited from different parents are both present but can be of different magnitude. To allow for multiple testing, we adjusted the p-value threshold for statistical significance by the effective number of independent tests^26^ performed, which takes account of the correlations (i.e., LD) between SNPs. Lastly, we attempted to replicate the findings of epistasis on an independent GWAS dataset of Korean populations.

## Results

### Marginal association analysis

We first tested the marginal association between HSCR and the common variants (minor allele frequency (MAF) > 5%) within the TADs of *RET* as well as *NRG1*. The variant rs2506006 (chr10:43581501) in *RET* and rs7005606 (chr8:32401501) in *NRG1* were identified as the most significant variants in these two regions (Table 1). The variant rs2506006 in *RET* is in nearly perfect LD (*r*^2^ = 0.98) with the lead variant rs2435357 that was found in previous study^8^. In the following analyses, we considered rs2435357 and rs7005606 as the lead SNPs for *RET* and *NRG1* loci, respectively. Rs2435357 was used instead of rs2506006 as rs2435357 has been much more studied^27,28^, both statistically and biologically, and was shown to have strong enhancer activities that regulate the expression of *RET*. Conditional association tests of other SNPs in the *RET* and *NRG1* TADs did not find other significant marginal effects after adjusting for the corresponding lead SNPs.

**Table 1:** Top variants that have strong marginal associations in *RET* and *NRG1*.

Rs ID	Variant	MAF	*p*-value
***RET***			
rs2506006	chr10:43581501:A:G	0.35	9.24E−43
rs2435357	chr10:43582056:T:C	0.35	1.66E−42
rs2435359	chr10:43580015:A:G	0.35	1.66E−42
***NRG1***			
rs7005606	chr8:32401501:T:G	0.28	1.72E−09
rs6996585	chr8:32400803:A:G	0.28	2.88E−09
rs10090954	chr8:32402501:C:A	0.48	4.85E−08

*rs2506006 is in high linkage disequilibrium with rs2435357 (*r*^2^ = 0.98).

Variant column has the format of chromosomal position: alternative allele: reference allele.

### Epistasis and secondary epistasis analysis

The focused regions (*RET* and *NRG1*) have been completely phased. We then detected epistatic effects between the lead variants (rs2435357 in *RET* and rs7005606 in *NRG1*) and all other common variants within the TADs of *RET* and *NRG1*. We considered four epistasis models (3)–(6) to explore different interaction mechanisms: phase-independent model, *cis* model, *trans* model, and C&T model. Briefly, the phase-independent model considers the interactions through the standard genotype effects, while the other three models incorporate the haplotype effects where the risk alleles inherited from the same or different parents can be tested (the more details can be found in the “Methods” section).

**Figure 1:**
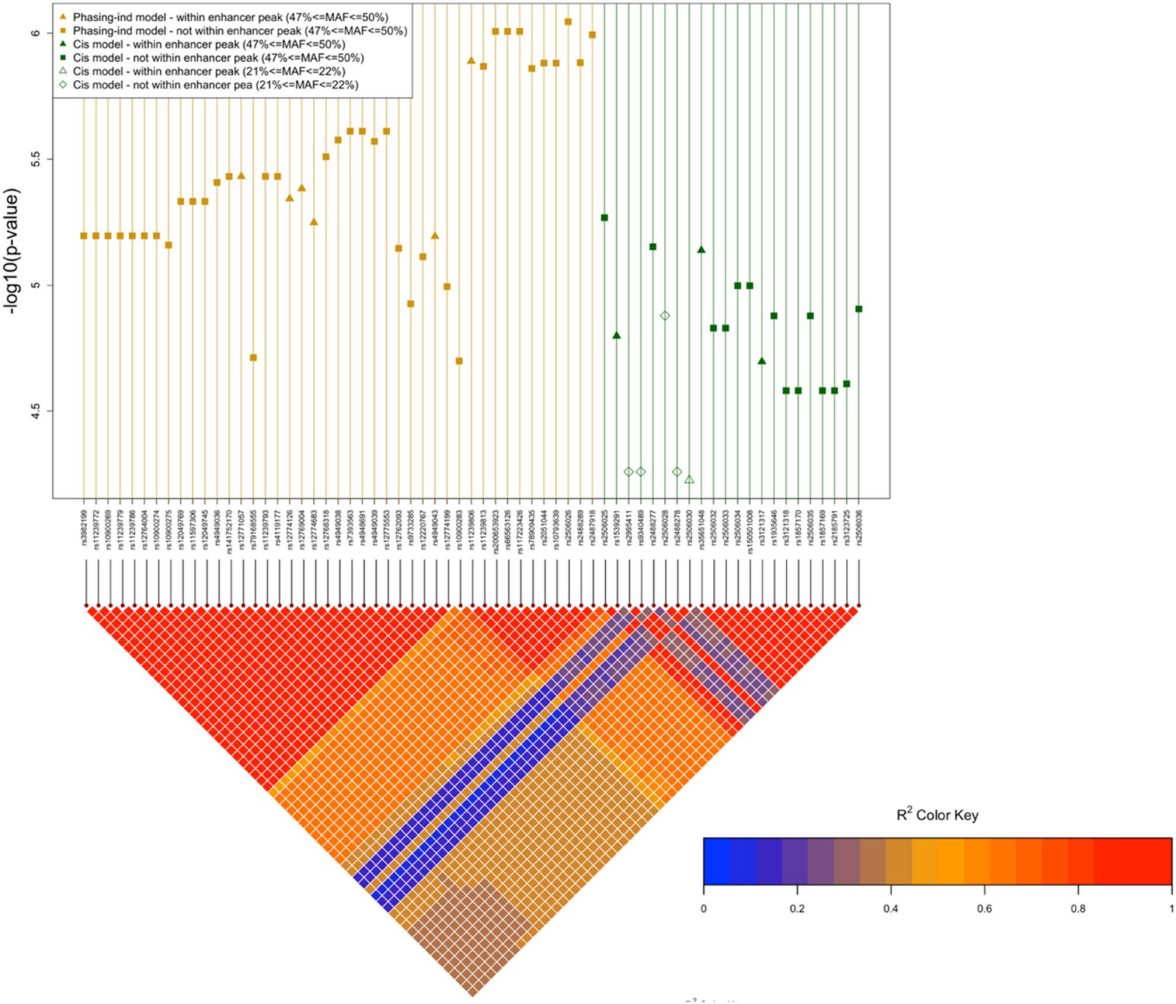
The LD pattern and − log10 (p-value) for 65 significant epistatic variants identified in *RET.* The 65 significant epistatic variants are those identified as significant (p < 0.05/727) based on at least in one epistasis model (phasing/phase-independent, cis, trans and C&T). The − log(p-value) are the log of the most significant p-values obtained for those 65 epistatic variants across epistasis models. The variants are ordered based on the physical location.

We adopted the Bonferroni correction method to adjust the p-values in the multiple testing. Realizing that the tests we performed in the study may not be independent due to the LD between SNPs, we used an estimated effective number of independent tests in the Bonferroni correction^26^. Using the method from Li et al., we estimated that the effective number of independent tests is 727 for the *RET* locus, and 590 in the *NRG1* locus, which corresponds to the p-value thresholds of p < 0.05/727 = 6.9 × 10^–5^ and p < 0.05/590 = 8.5 × 10^–5^ in *RET* and *NRG1*, respectively. We used these two thresholds throughout this study to identify the significant signals. The evidence of epistasis was found in *RET* using the phase-independent, cis, and C&T models but not by the trans model. Specifically, we successfully identified 48 epistatic variants using the phase-independent model, 33 epistatic variants using the cis model, and 53 epistatic variants using the C&T model (Supplemental Tables S1–S3). Amongst them, 65 unique variants were identified to have the significant epistasis effect with the lead variant with at least one epistasis model (see the summary in Fig. 1 and Supplemental Table S4). In order to identify independent signals among the detected epistasis variants in *RET*, a secondary epistasis analysis was conducted by conditioning each detected epistasis variant from the marginal association tests on the most significant epistasis variant (more details can be found in the Supplemental methods). The results from secondary epistasis analysis revealed that all detected epistasis variants by different epistasis models do not have independent roles, which might be driven by the high LD between the epistasis variants with the top epistasis variants (Supplemental Fig. S2). However, a subset of the significant epistasis variants with a different MAF range (21–22%) from that of the majority (46–49%), and for these variants the evidence for epistasis was strongest under the cis model (Fig. 1 and Supplemental Fig. S2; Supplementary Table S2). This suggests the potential independence between these two clusters. The secondary epistasis analysis did not identify significant independent effects of the second cluster of variants, which possibly due to inadequate statistical power.

**Figure 2:**
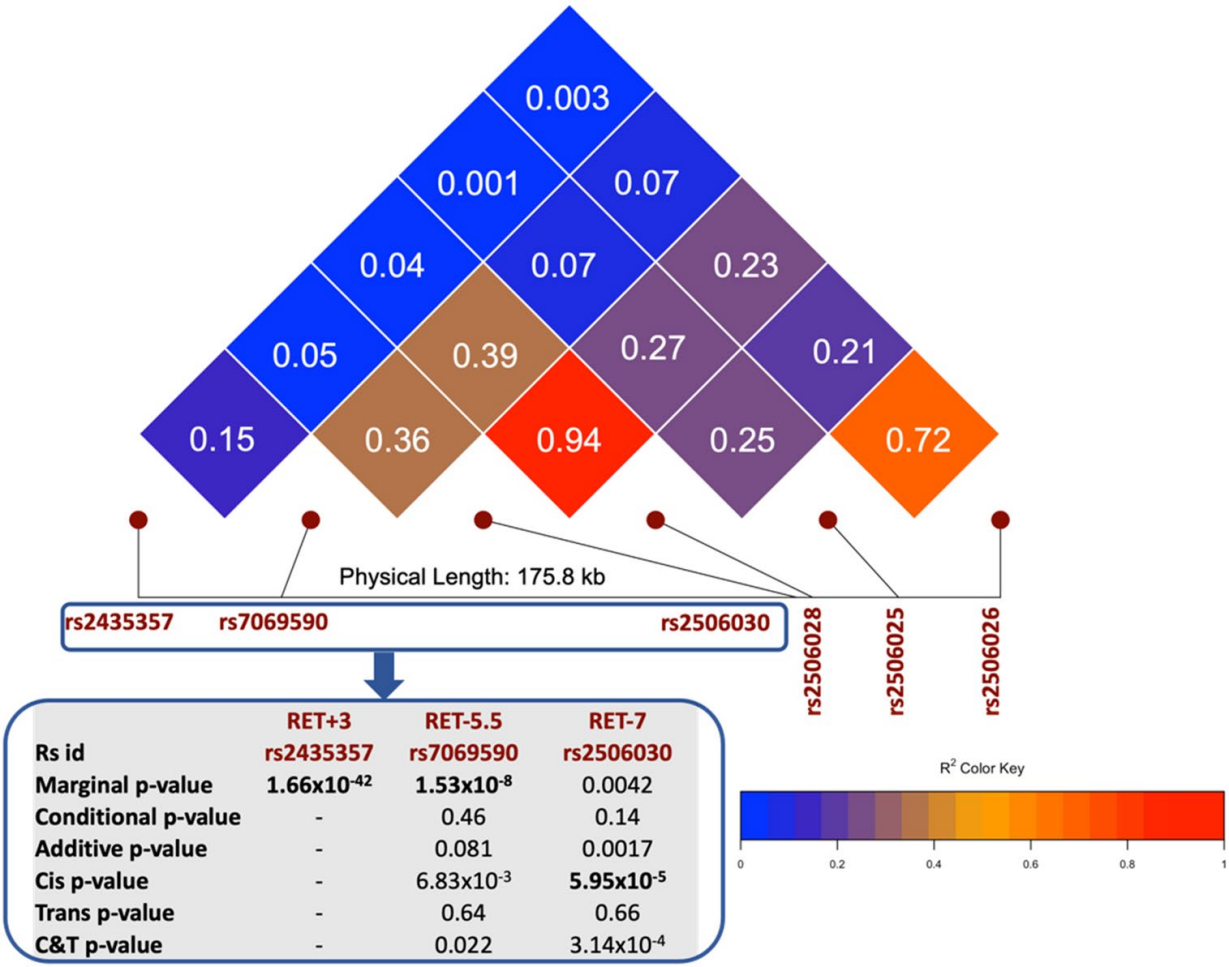
LD measures among three SNPS (rs2506030 (*RET-7*), rs7069590 (*RET-5.5*), rs2435357 (*RET*+*3*)) and the three representative epistatic variants (rs2506026, rs2506025, rs2506028). The conditional p-value refers to the p-value for the analysis conditioning on the lead variants; the additive p-value refers to the p-value obtained from the phasing-independent model.

Figure 1 shows the 65 potential epistastic variants detected by any of the four epistasis models (3)–(6), where the LD and the best p-value obtained in the epistasis analysis are summarized. The phase-independent model and the *cis* model provided more significant results than the C&T model for the commonly detected variants. In the figure, the epistatic variants are ordered based on the physical position on the chromosome. We can see that the phase-independent model performs best more often than other models. The high LD and similar p-values within the same cluster of variants as shown in the LD plot made it less possible to identify the true causal epistatic variants in the current analysis. Thus, we took the epistatic variants from each cluster/model with the smallest p-value as the representative variants. The most significant epistatic variants we found are rs2506026 (p-value = 9.01 × 10^–7^ in phase-independent model, p-value = 2.40 × 10^–6^ in C&T model; MAF = 0.47) and rs2506025 (p-value = 5.39 × 10^–6^ in cis model; MAF = 0.49). We noted that both the detected top epistasis variants (rs2506025 and rs2506026) do not fall inside an enhancer peak region as defined using ChIP-seq and ATAC-seq data from the disease-relevant enteric neural crest cells differentiated from induced pluripotent stem cells^29^. Within the enhancer peaks around *RET*, rs35651048 is the most significant variant using the cis model (*p* = 7.3 × 10^–6^) and rs11239806 is the top epistatic variant detected by the phase-independent model (*p* = 1.3 × 10^–6^) and C&T model (*p* = 5.0 × 10^–6^). While the top epistatic variants (rs2506025 and rs2506026) we detected are not within the enhancer peak region, they are in high LD with rs35651048 and rs11239806 (*r*^2^ = 0.85 and 0.98 respectively), which indicates that their epistatic effect may act through the enhancer variants. Moreover, among the 33 epistatic variants that pass the p-value threshold using cis model, the variant rs2506030 (*RET-7,* MAF = 0.22, *p* = 6 × 10^–5^) reported to have active regulatory roles in the expression of the *RET* gene in Chatterjee et al*.*^4^ is identified to have the strong interaction with the lead variant rs2435357 and mapped to the second cluster of *cis*-epistatic variants with lower range of MAF. The other variant rs7069590 (*RET-5.5*) highlighted in their study shows a significant marginal association (p = 9.01 × 10^–8^) but this variant is no longer marginally significant after conditioning on the lead variant (see Fig. 2).

None of the variants that have significant interaction effects with the lead variants in *NRG1* were detected in our dataset (Supplemental Table S5). Moreover, we also examined evidence for statistical epistasis between *RET* and *NRG1*: all variants within *RET* were examined for epistasis with rs7005606 in *NRG1* using the phase-independent model and likewise variants within *NRG1* with rs2435357 in *RET*. Only the phase-independent model that ignores the haplotype effects was accessed, as the phasing process is not accurate across different chromosomes. This analysis did not show the epistasis effect between *RET* and *NRG1* (Supplemental Table S6).

**Figure 3:**
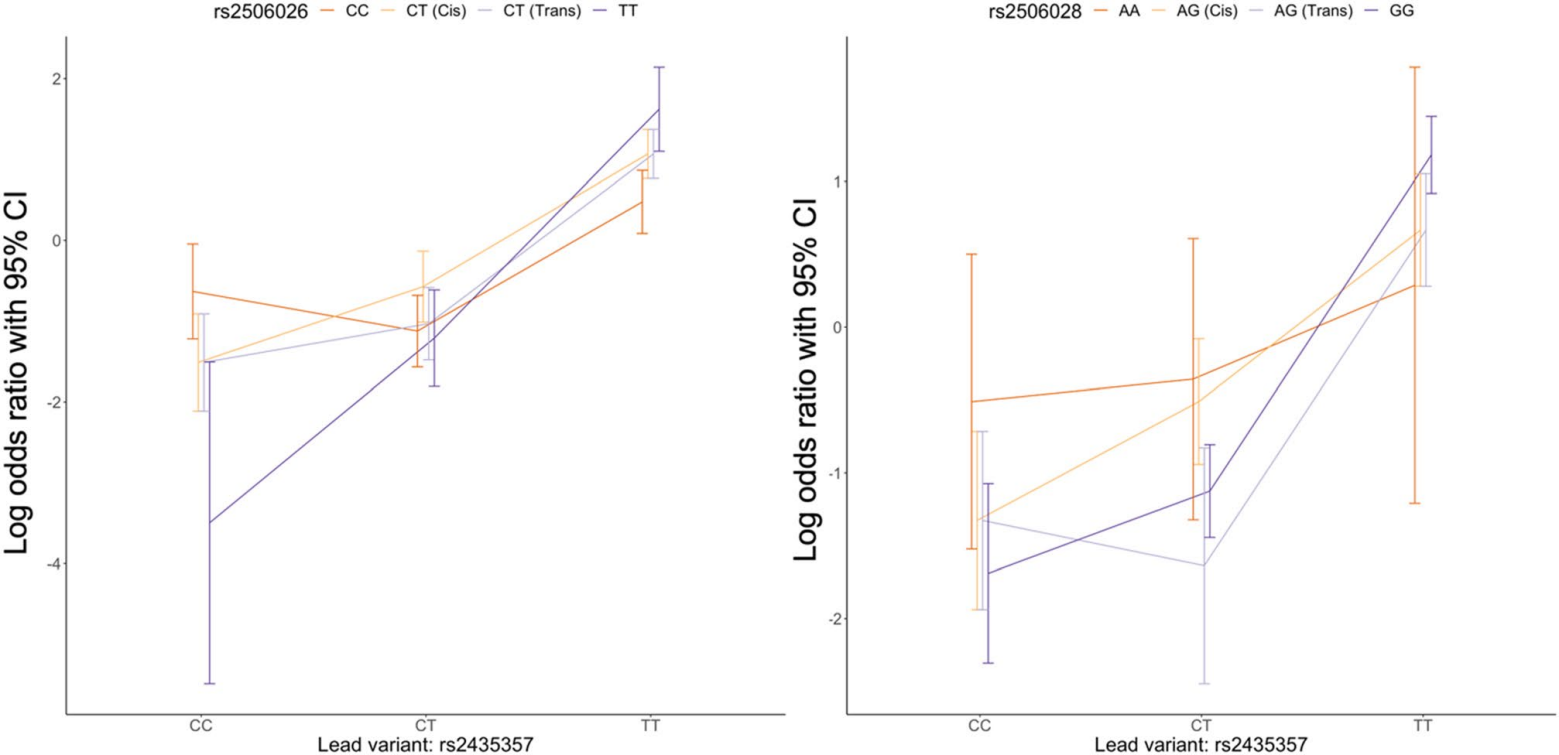
The epistatic effects in log odds between the two representative epistatic variants with the lead variant (rs2435357) in *RET.* On the left panel, CT (Cis) refers to the scenario where the risk allele (T) in rs2506026 and the risk allele (T) in rs2435357 are inherited from the same parent; CT (Trans) refers to the scenario where the risk allele (T) in rs2506026 and the risk allele (T) in rs2435357 are inherited from the different parents; Similarly, on the right panel AG (Cis) refers to the scenario where the risk allele (A) in rs2506028 and the risk allele (T) in rs2435357 are inherited from the same parent; AG (Trans) refers to the scenario where the risk allele (A) in rs2506028 and the risk allele (T) in rs2435357 are inherited from the different parents.

Figure 3 summarizes a closer examination of the representative epistatic variants (rs2506026 and rs2506028) with rs2435357 graphically. A clear upward trend of increasing log odds ratios can be seen going from the CC to CT to TT genotype in the lead variant (rs2435357). The genotypes in rs2435357 yield different effect of genotypes in rs2506026 on the odds of HSCR: when rs2435357 is CC, the C allele (vs. T) in rs2506026 appears to increase the risk of HSCR, while the C allele in rs2506026 is associated with a decreased risk when rs2435357 is TT. When rs2435357 is CT, the heterozygous genotype in rs2506026 leads to a higher risk of HSCR. The CT genotypes are divided into two groups, in which CT (Cis) happens when the risk allele T and the risk allele T in rs2435357 are inherited from the same parent, and CT (Trans) happens when the two risk alleles are inherited from different parents. It shows that the CT (Cis) always has equally or higher effect on the disease risk than the CT (Trans). The overall patterns in log odds suggests a clear additive interaction between rs2506026 and rs2435357. A similar trend is seen in the relationship between the genotypes in rs2435357 and rs2506025 that is identified using the cis model (Supplemental Fig. S3) given the moderate LD between them. The difference in log odds for CA (Cis) and CA (Trans) seems larger when there is a presence of heterozygous genotype in rs2435357, and such non-additive relationships could be further explained by the cis or trans interaction mechanisms. We also visualized the log odds among different genotypes in lead variant (rs2435357) and the other representative epistatic variant rs2506028 (the epistatic variant from cis model that represents the second cluster with a MAF ~ 22%). In contrast, the right panel in Fig. 3 shows an overall smaller change in log odds for rs2506028 among different genotypes in rs2435357 compared to the left panel, and it seems that the A allele in rs2506028 always increases the disease risk regardless of the genotypes in the lead variant, which all suggests a weaker additive epistasis effect. The AG (Cis) and AG (trans) yield the similar effect on log odds when the genotypes in lead variants are homozygous, while the significant log odds difference in the AG (Cis) and AG (trans) is observed when the lead variants is CT and such non-linear effect was captured using the cis model through the haplotype effects.

### Replication analysis in Korean data

Next, we attempted to replicate the epistatic effects detected in the *RET* locus on an independent GWAS dataset of Korean populations (122 cases and 374 controls)^30^ as no other WGS analysis has been conducted on HSCR thus far. Compared to the findings in Chinese data described above, the similar inheritance patterns in the lead variants were observed in the Korean data. The *RET* lead variant (rs2435357) has the dominant inheritance pattern as suggested by the underlying odds ratios within in three different genotypes (Table S7), however the dominant effect did not pass the significance level in the standard Likelihood Ratio Test (LRT) (p = 0.27) due to the limited power. Moreover, the additive inheritance pattern was observed in *NRG1* lead variant (rs7005606).

Three variants (rs2506026, rs7393563, and rs2506025) were chosen as the best LD proxies to represent and account for the missingness of the 48 epistatic variants previously identified using the phase-independent model in the Chinese data. As shown in the Fig. S5, the three proxy variants can well represent the three LD blocks of the 48 epistatic variants. Based on the phase-independent model, the proxy variant rs2506034 representing the small group of 5 epistatic variants showed a significant interaction with the lead variant (p = 0.03), replicating the observed epistasis detected in the Chinese WGS data (Figs. S5, S6; Table S8), where Fig. S6 suggests that the epistasis patterns are generally similar in the two datasets. For the three most significant epistatic variants (rs2506026, rs7393563, and rs2506025) in the Chinese dataset that highly correlate to these three proxy variants, larger standard error and wider confidence interval in the estimates were observed in the Korean data (Table S8), which also suggests a power issue due to the smaller sample size. Lastly, the model with the additional adjustment of epistasis effect showed an improvement in heritability (~ 1%) on top of the model with only the main effect (see details in the Supplemental methods).

## Discussion

In this project, we performed an exploratory analysis to look for statistical epistasis in two Hirschsprung disease related regions of the genome—the TADs encompassing the *RET* and *NRG1* genes. Our epistasis analysis identified the variants in 130–150 kb upstream of *RET* that appeared to show epistatic effects with the lead variant in the region: rs2506026 and rs2506025. Significant epistatic effects were found using the phase-independent, cis, C&T epistasis models, where the phase-independent model models the interactions between genotypes, and cis model models the interactive effects among two risk alleles inherited from the same parent by incorporating the haplotype effect, and C&T models captures the combined cis and trans effects (risk alleles inherited from different parents). The stronger epistatic effect identified using the phase-independent model with the better fit suggests that haplotype effects alone were not sufficient to explain the epistasis. Other mechanisms, such as autoregulation and positive/negative feedback from the gene regulatory network, may also be involved in the epistatic interaction. It has been pointed out in the literature^31^ that apparent statistical epistatic effects could be due to haplotype tagging of an untyped SNP. Here, we used WGS data and avoided the confounding by assaying nearly all variants, except for complex cases such as tandem repeats. The fact that the additive epistasis effect was more significant also argues against the interpretation that this effect was due to an untyped SNP.

The epistasis analysis was done by evaluating the interaction effects between the lead variant and other variants within the *RET* and *NRG1* regions. We used rs2435357 as the lead variant in *RET*, and rs7005606 as the lead variants in *NRG1*. After exploring the different interaction mechanisms, we detected the omniums set of 65 significant epistatic variants that pass the significance threshold (p < 0.05/728) for *RET* using at least one epistasis model. It has also been shown that, among the top epistatic variants, the phase-independent model provides a better performance in terms of AIC/BIC, and the phase-independent or cis models always yield more significant p-values for the commonly detected variants than the C&T. The secondary epistasis analysis revealed that all the detected epistatic variants do not have independent roles to contribute the epistasis effects on HSCR. However, the LD patterns in Fig. 1 suggests a possibly independent effects of the two clusters of epistatic variants. We also want to note that the similar significance and strong or moderate LD were observed within these two clusters of epistatic variants. which implies two independent causal variants (from the two clusters) with the true epistasis effects exist and the remaining variants in the omniums set were detected might due to the underlying LD. One limitation in our study is that it is less possible to identify the true causal epistatic variants statistically among LD proxies even with the WGS and other annotation information will be needed to further implicate the functional regulatory epistasis variants. Instead, we took the top variants from each cluster as the representative epistatic variants: rs2506026 and rs2506028. Their overall trends in log odds among different genotypes in lead variant provide the consistent evidence on the interaction mechanisms as revealed by phase-independent, cis or C&T models. We were not able to detect the epistatic variants that have strong interactions with the lead variant within *NRG1* or between *RET* and *NRG1* due to the limited cases that carry the risk alleles in *NRG1*. The findings of additive epistasis effects (using the phase-independent model) were replicated using the Korean data, where we showed a significant interaction effect through the proxy variant rs2506034 that well represents the small group of 5 epistatic variants based on the LD. In the Chinese analysis, these 5 epistatic variants (rs2506025, rs2488277, rs1539291, rs35651048, and rs2506036) were all identified by the phase-independent, cis, and C&T models, but the results showed that they have the most significant p-values with the Cis model (Fig. 1), which potentially suggests a non-linear effect between the variants and the disease risk and such effect is more closed to Cis interactive mechanism. Moreover, we also presented a gain in heritability in the model with the additional inclusion of epistasis effect in comparison to the model without the epistasis effect, although the improvement was not very significant given the low statistical power in the Korean data. We also want to note the small sample sizes in both discovery and replication data may cause the false positive results. Currently the WGS data on non-Asian samples are not available yet. In the future, we hope to generate WGS from more samples to further replicate those results.

The roles of rs2435357 in *RET* and rs7005606 in *NRG1* have been confirmed by previous study. Moreover, rs7005606 was also the lead variant identified in the recent meta-analysis by^2^, which is correlated with the previous lead variant for *NRG1* rs7835688^10,32^ with r^2^ = 0.70. Rs9282834 (MAF = 2.3% in our study), the previously identified conditionally significant variant^2^, did not show any significance in all epistasis analyses we considered. The lead variant rs2435357 in *RET* was highlighted with other two variants in Chatterjee et al.^4^. They also provided evidence for the regulatory roles of these three variants, with *RET-7* (rs2506030), *RET-5.5* (rs7069590), and *RET*+*3* (rs2435357) being the transcription factor binding sites essential for enteric nervous system (ENS) development^27^. We summarized the statistical significance of the other two SNPs (rs2506030 and rs7069590) highlighted in their paper, with respect to the marginal, conditional, and epistatic associations (Fig. 2). *RET-5.5* (rs7069590) is significantly associated to HSCR in the marginal association, and *RET-7* (rs2506030) shows a significant cis epistasis effect. *RET-7* (rs2506030) is bound by the retinoic acid receptor beta (Rarb) and the risk variant was shown to reduce Ret expression through reducing binding of Rarb and hence the enhancer activity. While *RET*+*3* (rs2435357) has been well established as a risk variant in HSCR and the lead SNP of this study, we were not able to find evidence for statistical epistasis between *RET-5.5* (rs7069590) and *RET*+*3* (rs2435357). Moreover, there is no evidence of independent association between *RET-5.5* (rs7069590) and HSCR after accounting for LD with *RET*+*3* (rs2435357). Thus, despite evidence for independent biological functions of three *RET* variants in regulating its expression, this does not translate to statistical independence. Our study suggests a more prominent role of the *RET*+*3* (rs2435357) variant alone, with possible modulatory effects by *RET-7 (*rs2506030*)*, and no important contribution from the *RET-5.5* variant (rs7069590). Our finding on epistasis on *RET* could be generalized to other populations, such as European population, as the previous biological evidence on epistasis has been shown in Chatterjee et al. in which the variants tested were prioritized from GWAS on European populations^19^. However, because of the shortage of the WGS in other populations, we can only study Asian population now. When available, WGS data on other populations will help fine map the causal epistatic variant(s) that may modify the penetrance for HSCR.

Considering that the patients with high penetrant, pathogenic mutations in *RET* and other known HSCR genes might affect our results, we performed additional epistasis analysis by removing 5 samples with rare, loss of function (LoF) variants in *RET* and 3 syndromic cases with LoF variants in *ZEB2* based on function annotations using KGGSeq*.* We identified the same 61 unique epistatic variants with the same best model compared to our results that use all 936 samples (Table S9). Because of the smaller sample size, four previously identified variants (rs2995411, rs9340489, rs2488278, rs2506030) did not pass the significance threshold and the determined variants have less significant p-values after excluding 8 samples.

In summary, our results offer the statistical evidence for the epistasis effect between the genetic variations upstream of the promoter with the lead variant in *RET*, and such effect might act through the enhancer variants suggested by the high LD between the top epistatic variants with the top epistatic variants within the enhancer peaks. Given that HSCR is found to be contributed only by a small number of common variants but with high heritability, our findings can provide more insights on the contribution of epistasis effects on the unexplained heritability in the HSCR.

## Materials and methods

### Study samples

The discovery cohort comprised 443 short segments HSCR (S-HSCR) cases and 493 controls analyzed by WGS and passed the quality control (as described in Tang et al.^24^). All patients analyzed were sporadic, with no known family history of HSCR, and were recruited at hospitals in China (n = 341) and Vietnam (n = 102). To minimize confounding due to population stratification, controls were ascertained from the same or nearby cities to match with the subpopulations of the patients. Informed consent was obtained from all participants and the study was approved by the institutional review board of the University of Hong Kong and the Hospital Authority. The research was conducted performed in accordance with the Declaration of Helsinki.

To replicate the analysis, we used the Korean GWAS data of sporadic HSCR cases and controls. In the replication analysis, we only included the unrelated samples from 122 cases and 374 controls. The proxy variants were used to the represent the missing variants in the Korean dataset, based on their underlying LD.

### Genotype data

The discovery sample was whole genome sequenced using Illumina HiSeq X Ten to a mean coverage of 30×. Raw sequence reads were first aligned to human reference genome (hg19) using Burrows-Wheeler Aligner (BWA-MEM). Aligned reads were then processed according to Genome Analysis Toolkit (GATK; version 3.4) and which resulted a final call set of 33.4 million (M) single nucleotide variants (SNVs) and 3.3M indels. To determine the haplotype configurations for variants in *RET* and *NRG1*, we performed read aware phasing using SHAPEIT2. Briefly, phase informative reads spanning at least two heterozygous sites were obtained from the BAM files and were used to phase the common and rare variants in VCF file.

### Topologically associating domains and enhancer peaks

TADs are defined using the TADs called from Hi-C data of 9 cell lines from Rao et al.^33^. The union of the different intervals across cell types is used, which results in the 2.2 Mb and 2.5 Mb TAD regions for *RET* (chr10:43340000–45560000) and *NRG1* (chr8:31070000–33600000), respectively. We extended the region of interest to chr10:43000000–45560000 (2.6 Mb) for *RET* to cover more significant variants for *RET*. In our paper, we use the same enhancer list from^29^ to prioritize the variants within the enhancer peak.

### Marginal association and conditional analyses

Marginal variant association tests were performed using the following logistic regression assuming the additive and dominant genetic effects:

1 $$ {\text{logit}}\;p_{i} = \alpha_{0} + \alpha_{1} PC1i + \alpha_{2} PC2i + \gamma_{1} A_{i}^{j} + \gamma_{2} D_{i}^{j} , $$

where $A_{i}^{j}$ and $D_{i}^{j}$ are the additive and dominant effects for variant j that satisfies the selection criteria within the region for sample i. Principal Component Analysis (PCA) was performed using PC-AiR^34^ to control for the population structure (the details of PCA can be found in the Supplemental methods). We adjusted for population stratification using the first two PCs (i.e., *PC*1 and *PC*2). Tests were limited to common variants with MAF of at least 0.05 in the data. Significance level was assessed using the Likelihood Ratio Test (LRT) where we compared the model with the genetic effect with the null model (only contains the covariates). Altogether 6810 and 4779 variants and short indels were considered within *RET* and *NRG1* respectively.

To detect the variants beyond the lead variants (we used rs2435357 in *RET* and rs7005606 in *NRG1* in this paper), a series of conditional analyses for the significant epistatic variants from marginal tests were then conducted by conditioning on the lead variants within the *RET* and *NRG1* regions, i.e.,

2 $$ {\text{logit}}\;p_{i} = \alpha_{0} + \alpha_{1} PC1i + \alpha_{2} PC2i + \gamma_{1} A_{i}^{j} + \gamma_{2} D_{i}^{j} + \gamma_{3} A_{i}^{lead} + \gamma_{4} D_{i}^{lead} , $$

where $A_{i}^{lead}$ and $D_{i}^{lead}$ are the additive and dominant effects for the lead variants detected through the marginal association analysis. The significance for variant j was evaluated through the LRT that compares the model (2) and model (1).

### Epistasis models

We accessed statistical epistasis by testing the variants within the *RET* and *NRG1* with their corresponding lead variants, where the logistic regression is applied. To test the models with different interactive mechanisms among risk alleles, the following four epistasis models are proposed:

***Phase-independent***

3 $$ {\text{logit}}\;p_{i} = \alpha_{0} + \alpha_{1} PC1i + \alpha_{2} PC2i + \gamma_{1} A_{i}^{lead} + \gamma_{2} D_{i}^{lead} + \gamma_{3} A_{i}^{j} + \gamma_{4} D_{i}^{j} + \beta_{1} A_{i}^{lead} A_{i}^{j} $$

***Cis***

4 $$ {\text{logit}}\;p_{i} = \alpha_{0} + \alpha_{1} PC1i + \alpha_{2} PC2i + \gamma_{1} A_{i}^{lead} + \gamma_{2} D_{i}^{lead} + \gamma_{3} A_{i}^{j} + \gamma_{4} D_{i}^{j} + \beta_{1} (H_{i}^{lead,1} H_{i}^{j,1} + H_{i}^{lead,2} H_{i}^{j,2} ) $$

***Trans***

5 $$ {\text{logit}}\;p_{i} = \alpha_{0} + \alpha_{1} PC1i + \alpha_{2} PC2i + \gamma_{1} A_{i}^{lead} + \gamma_{2} D_{i}^{lead} + \gamma_{3} A_{i}^{j} + \gamma_{4} D_{i}^{j} + \beta_{1} (H_{i}^{lead,1} H_{i}^{j,2} + H_{i}^{lead,2} H_{i}^{j,1} ) $$

***C&T***

6 $$ {\text{logit}}\;p_{i} = \alpha_{0} + \alpha_{1} PC1i + \alpha_{2} PC2i + \gamma_{1} A_{i}^{lead} + \gamma_{2} D_{i}^{lead} + \gamma_{3} A_{i}^{j} + \gamma_{4} D_{i}^{j} + \beta_{1} (H_{i}^{lead,1} H_{i}^{j,1} + H_{i}^{lead,2} H_{i}^{j,2} ) + \beta_{2} (H_{i}^{lead,1} H_{i}^{j,2} + H_{i}^{lead,2} H_{i}^{j,1} ) $$

where $A_{i}^{j}$ and $D_{i}^{j}$ denote the additive and dominant effect respectively of variant *j* for sample *i*, and $H_{i}^{j,1}$ and $H_{i}^{j,2}$ denote the haplotypes for the same variants, with $H_{i}^{j,1} + H_{i}^{j,lead} = A_{i}^{j}$. $A_{i}^{lead} ,\;D_{i}^{lead} ,\;H_{i}^{lead,1} ,H_{i}^{lead,2}$ denote the equivalent for the lead variant, and *p*_*i*_ is the probability of having the disease. The statistical significance for the epistasis effect is obtained through the LRTs by comparing each epistasis model with the base model defined as following:

$$ {\text{logit}}\;p_{i} = \alpha_{0} + \alpha_{1} PC1i + \alpha_{2} PC2i + \gamma_{1} A_{i}^{lead} + \gamma_{2} D_{i}^{lead} + \gamma_{3} A_{i}^{j} + \gamma_{4} D_{i}^{j} , $$

which corresponds to the chi-square test with 1 degree of freedom when compare additive, cis, and trans models to the base model, and the chi-square test with 2 degrees of freedom when compare the C&T model to the base model.

### Replication analysis using Korean data

The Korean data was used to replicate our specific findings of epistasis effects with the phase-independent model. Recall that we identified 48 significant epistatic variants using the same model in the Chinese dataset, however, because of their high LDs and similar p-values, we could not identify the exact causal variant with the true epistasis effect. Therefore, in the replication analysis, we considered all the 48 epistatic variants in the epistasis analysis. In the Korean data, rs2505998 was used as a proxy for the *RET* lead variant which was not genotyped in the GWAS data, and the variants (rs2487918, rs11239767, rs2506034) were used as the three best proxy variants that can well represent the 48 epistatic variants based on the LDs. More detailed analysis can be found in the Supplemental methods.

## Supplementary Information

Supplementary Tables.

Supplementary Information.

## Data Availability

The datasets analysed for the current study are available upon request to the corresponding author (C.S.T.).
