# Supplementary material for: Whole genome sequencing reveals epistasis effects within RET for Hirschsprung disease
Source: Sci Rep. 2022 Nov 28;12:20423. doi: 10.1038/s41598-022-24077-w (PMC9705416; doi:10.1038/s41598-022-24077-w)
Supplement: Supplementary file 2 — Supplementary Information. [file 41598_2022_24077_MOESM2_ESM.docx]

**Supplemental methods**

***Principal component analysis***

The Principal Component Analysis (PCA) was performed using PC-AiR [Conomos et al. 2015] to visualize and understand the population structure in the dataset. The variants that pass the threshold of MAF > 5%, call rate > 95%, linkage disequilibrium (LD) with a threshold of squared correlation (R2) < 0.1 in sliding 200KB window, and the Hardy-Weinberg Equilibrium (HWE) p-value > 10^-6^ in cases were involved in the analysis. From the PC plot that is color-coded by the residencies where the samples were collected (Supplemental **Figure S1**), we showed that PC1 can explain the geographic difference while PC2 explains the ancestry difference. We adjusted the top two PCs as the covariates in all statistical analysis.

***Secondary epistasis analysis in RET***

Based on the epistasis analysis, we detected the significant set of epistatic variants using phasing-independent, cis, and C&T models. Because the identified significant epistatic variant could have independent role or non-independent role from other epistatic variants, the secondary epistasis analysis was then conducted.

We adopted a two-step procedure to 1) first detect the candidate variants that have independent role from main epistasis variants; and 2) identify the secondary epistasis variants. We denote “lead” as the lead variant (rs2435357) in *RET*, and j and k are the identified significant epistatic variants. For example, the following models are considered for the phasing-independent model that evaluates the interaction between genotypes:

**Secondary phasing-independent model** (var j is the main epistasis variants identified using the phasing-independent model)

$\mathrm{logit}p_{i} =\alpha_{1}PC1i+\alpha_{2}PC2i+{\gamma_{1}A}_{i}^{lead}+{\gamma_{2}D}_{i}^{lead}+{\gamma_{3}A}_{i}^{j}+\gamma_{4}D_{i}^{j}+{\gamma_{5}A}_{i}^{k}+{\gamma_{6}D}_{i}^{k}+{\beta_{1}A}_{i}^{lead}A_{i}^{j}+\beta_{2}A_{i}^{lead}A_{i}^{k},(1)$

**Secondary phasing-independent model – Base1**

$\mathrm{logit}p_{i} =\alpha_{1}PC1i+\alpha_{2}PC2i+{\gamma_{1}A}_{i}^{lead}+{\gamma_{2}D}_{i}^{lead}+{\gamma_{3}A}_{i}^{j}+\gamma_{4}D_{i}^{j}+\beta_{1}A_{i}^{lead}A_{i}^{j},(2)$

**Secondary phasing-independent model – Base2**

$\mathrm{logit}p_{i} =\alpha_{1}PC1i+\alpha_{2}PC2i+{\gamma_{1}A}_{i}^{lead}+{\gamma_{2}D}_{i}^{lead}+{\gamma_{3}A}_{i}^{j}+\gamma_{4}D_{i}^{j}+{\gamma_{5}A}_{i}^{k}+{\gamma_{6}D}_{i}^{k}+\beta_{1}A_{i}^{lead}A_{i}^{j}.(3)$

Then for Step1, the test is performed by comparing model (1) and model (2) and the significance is accessed using Likelihood Ratio Test (LRT) for the nested models; in Step 2, the LRT that compares model (3) and model (1) is proceeded. The significant secondary epistasis variants need to pass both Step 1 and Step 2 for all the 48 significant epistatic variants found from phasing-independent model. We follow the following steps to detect the significant secondary epistasis variants.


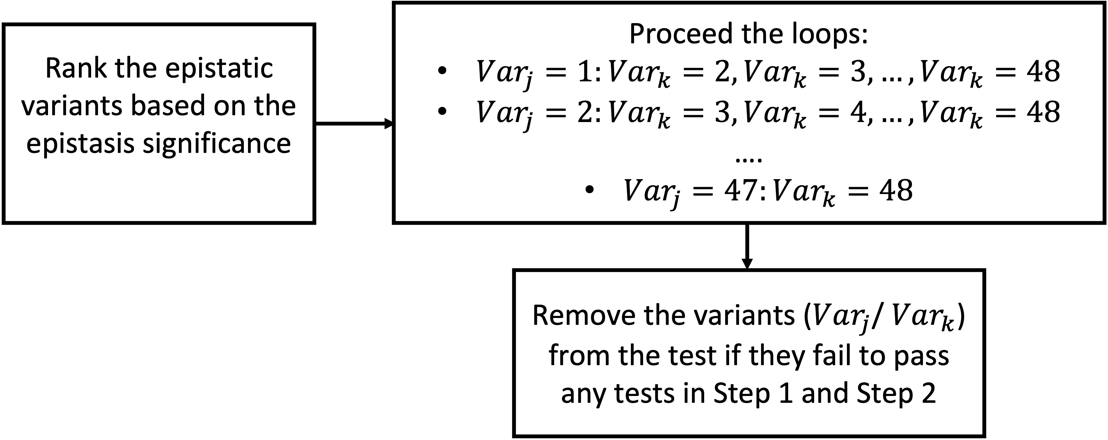


Similarly, we have the following secondary models for cis and epis interaction mechanisms:

**Secondary Cis** (var j is the main epistasis variants identified using the Cis model)

$\mathrm{logit}p_{i} =\alpha_{1}PC1i+\alpha_{2}PC2i+{\gamma_{1}A}_{i}^{lead}+{\gamma_{2}D}_{i}^{lead}+{\gamma_{3}A}_{i}^{j}+\gamma_{4}D_{i}^{j}+{\gamma_{5}A}_{i}^{k}+{\gamma_{6}D}_{i}^{k}+\beta_{1}\left( H_{i}^{lead,1}H_{i}^{j,1}+H_{i}^{lead,2}H_{i}^{j,2})+\beta_{2}{(H}_{i}^{lead,1}H_{i}^{k,1}+H_{i}^{lead,2}H_{i}^{k,2} \right),(4)$

**Secondary Cis – Base1**

$$\mathrm{logit}p_{i} =\alpha_{1}PC1i+\alpha_{2}PC2i+{\gamma_{1}A}_{i}^{lead}+{\gamma_{2}D}_{i}^{lead}+{\gamma_{3}A}_{i}^{j}+\gamma_{4}D_{i}^{j}+\beta_{1}\left( H_{i}^{lead,1}H_{i}^{j,1}+H_{i}^{lead,2}H_{i}^{j,2} \right),(5)$$

**Secondary Cis – Base2**

$$\mathrm{logit}p_{i} =\alpha_{1}PC1i+\alpha_{2}PC2i+{\gamma_{1}A}_{i}^{lead}+{\gamma_{2}D}_{i}^{lead}+{\gamma_{3}A}_{i}^{j}+\gamma_{4}D_{i}^{j}+{\gamma_{5}A}_{i}^{k}+{\gamma_{6}D}_{i}^{k}+\beta_{1}\left( H_{i}^{lead,1}H_{i}^{j,1}+H_{i}^{lead,2}H_{i}^{j,2} \right),(6)$$

**Conditional C&T** (var j is the main epistasis variants identified using the C&T model)

$\mathrm{logit}p_{i} =\alpha_{1}PC1i+\alpha_{2}PC2i+{\gamma_{1}A}_{i}^{lead}+{\gamma_{2}D}_{i}^{lead}+{\gamma_{3}A}_{i}^{j}+\gamma_{4}D_{i}^{j}+{\gamma_{5}A}_{i}^{k}+{\gamma_{6}D}_{i}^{k}+\beta_{1}(H_{i}^{lead,1}H_{i}^{j,1}+H_{i}^{lead,2}H_{i}^{j,2})+{\beta_{2}(H}_{i}^{lead,1}H_{i}^{k,1}+H_{i}^{lead,2}{H_{i}^{k,2}})+\beta_{3}\left( H_{i}^{lead,1}H_{i}^{j,2}+H_{i}^{lead,2}H_{i}^{j,1})+{\beta_{4}(H}_{i}^{lead,1}H_{i}^{k,2}+H_{i}^{lead,2}H_{i}^{k,1} \right) ,(7)$

**Conditional C&T – Base1**

$$\mathrm{logit}p_{i} =\alpha_{1}PC1i+\alpha_{2}PC2i+{\gamma_{1}A}_{i}^{lead}+{\gamma_{2}D}_{i}^{lead}+{\gamma_{3}A}_{i}^{j}+\gamma_{4}D_{i}^{j}+\beta_{1}\left( H_{i}^{lead,1}H_{i}^{j,1}+H_{i}^{lead,2}H_{i}^{j,2} \right)+\beta_{3}\left( H_{i}^{lead,1}H_{i}^{j,2}+H_{i}^{lead,2}H_{i}^{j,1} \right),(8)$$

**Conditional C&T – Base2**

$$\mathrm{logit}p_{i} =\alpha_{1}PC1i+\alpha_{2}PC2i+{\gamma_{1}A}_{i}^{lead}+{\gamma_{2}D}_{i}^{lead}+{\gamma_{3}A}_{i}^{j}+\gamma_{4}D_{i}^{j}+{\gamma_{5}A}_{i}^{k}+{\gamma_{6}D}_{i}^{k}+\beta_{1}\left( H_{i}^{lead,1}H_{i}^{j,1}+H_{i}^{lead,2}H_{i}^{j,2} \right)+\beta_{3}\left( H_{i}^{lead,1}H_{i}^{j,2}+H_{i}^{lead,2}H_{i}^{j,1} \right),(9)$$

Accordingly, the results in Step 1 can be obtained by LRT that compares model (5) vs. model (4) and model (8) vs. model (7) for cis and epis interaction mechanisms, respectively; and the results in Step 2 can be obtained by LRT that compares model (6) vs. model (4) and model (9) vs. model (7) for cis and C&T (cis and trans) interaction mechanisms, respectively. We follow the same procedure using the loops as we shown in the example using phasing-independent model to find the secondary epistatic variants among those 33 detected epistatic variants from cis models and 53 detected epistatic variants from C&T models (Supplemental **Tables S2-3**). We did not identify such secondary epistatic variants for all phasing-independent, cis and C&T models, suggesting that all the epistatic variants do not have independent roles for the trait.

***Results comparison with previous study***

Chatterjee et al. (2016) found from in vitro experiments that three SNPs near the RET gene (rs2506030 *(RET-7*), rs7069590 (*RET-5.5*), and rs2435357 (*RET+3*)) have active regulatory roles in the expression of the *RET* gene.

We compared the odds ratios derived in our study with those in Chatterjee's study (Supplemental **Figure S4**). It can be seen that the risk allele T in the SNP rs2435357 (*RET+3*) confers a significantly higher odds in all haplotypes, and the odds ratios are similar between our study and those of Chatterjee et al. (2016). The other haplotypes do not lead to increased risks of HSCR that were significantly different from the reference haplotype, except for the haplotype G*T*C in our study (haplotype with risk alleles of RET-7 and RET-5.5 and non-risk allele of RET+3), which show significant decreased risk of disease in their study.

***Replication analysis in Korean data***

The replication analysis was performed to replicate the findings, where the Korean data was used as the test data. Because the phasing-independent model is the most powerful model that identified most of the significant epistatic variants in the Chinese data, we tried to replicated these epistatic variants for the Korean data set with the phasing-independent model.

The dominant effects in the *RET* lead variant and *NRG1* lead variant were first tested by comparing the following two models

$$\mathrm{logit}p = \alpha_{0}+{\gamma_{1}A}^{lead},$$

$$\mathrm{logit}p = \alpha_{0}+{\gamma_{1}A}^{lead}+{\gamma_{2}D}^{lead}.$$

The p-values from the LRT suggest the insignificant dominant effects in these two lead variants (p= 0.27 for *RET* lead variant, p-vale = 0.99 *NRG1* lead variant). However, the dominant effect in *RET* lead variant was observed through the log odds ratio (**Table S7**).

Given that the dominant term for *RET* lead variant was not statistically significant after conditioning on the additive effect ${\gamma_{1}A}^{lead}$, the models with purely additive effect or the dominant effect were considered as the candidate base model for the epistasis analysis. The model with only the additive effect was then chosen as the base model as it has better AIC, i.e.,

$$\mathrm{logit}p = \alpha_{0}+{\gamma_{1}A}^{RET\_lead}+{\gamma_{3}A}^{RET\_epi\_var} (10),$$

and the significance for epistasis was accessed by comparing model (10) to the model

$$\mathrm{logit}p = \alpha_{0}+ {\gamma_{1}A}^{RET\_lead}+{\gamma_{3}A}^{RET\_epi\_var}+{\beta_{1}A}_{i}^{RET\_lead}A_{i}^{RET\_epi\_var} \left( 11 \right).$$

All three proxy variants that can represent the three groups of epistatic variants with the phasing-independent effects as shown in the LD plot (**Figure** **S5**) were tested, and amongst them, rs2506034 that represent the small group (including rs2506025, rs2488277, rs1539291, rs35651048, and rs2506036) shown an significant epistasis effect (p= 0.03). Compared to model (10), the model (11) with additional adjustment of epistasis effect revealed an improvement in heritability (~ 1%).

**Supplemental plots**


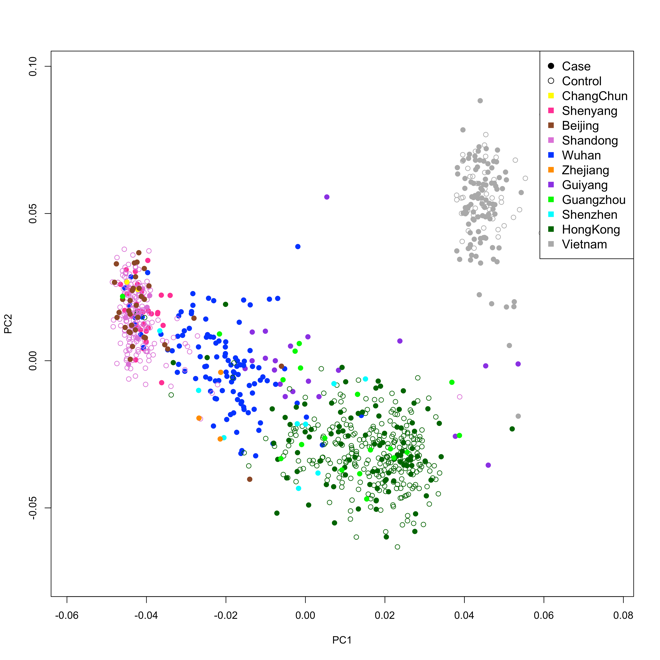

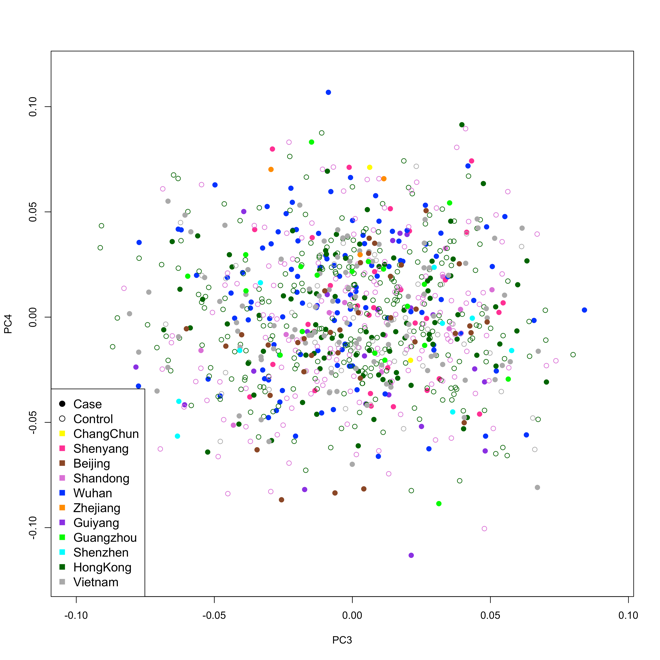


**Figure S1. PCA using S-HSCR cases and controls**

**
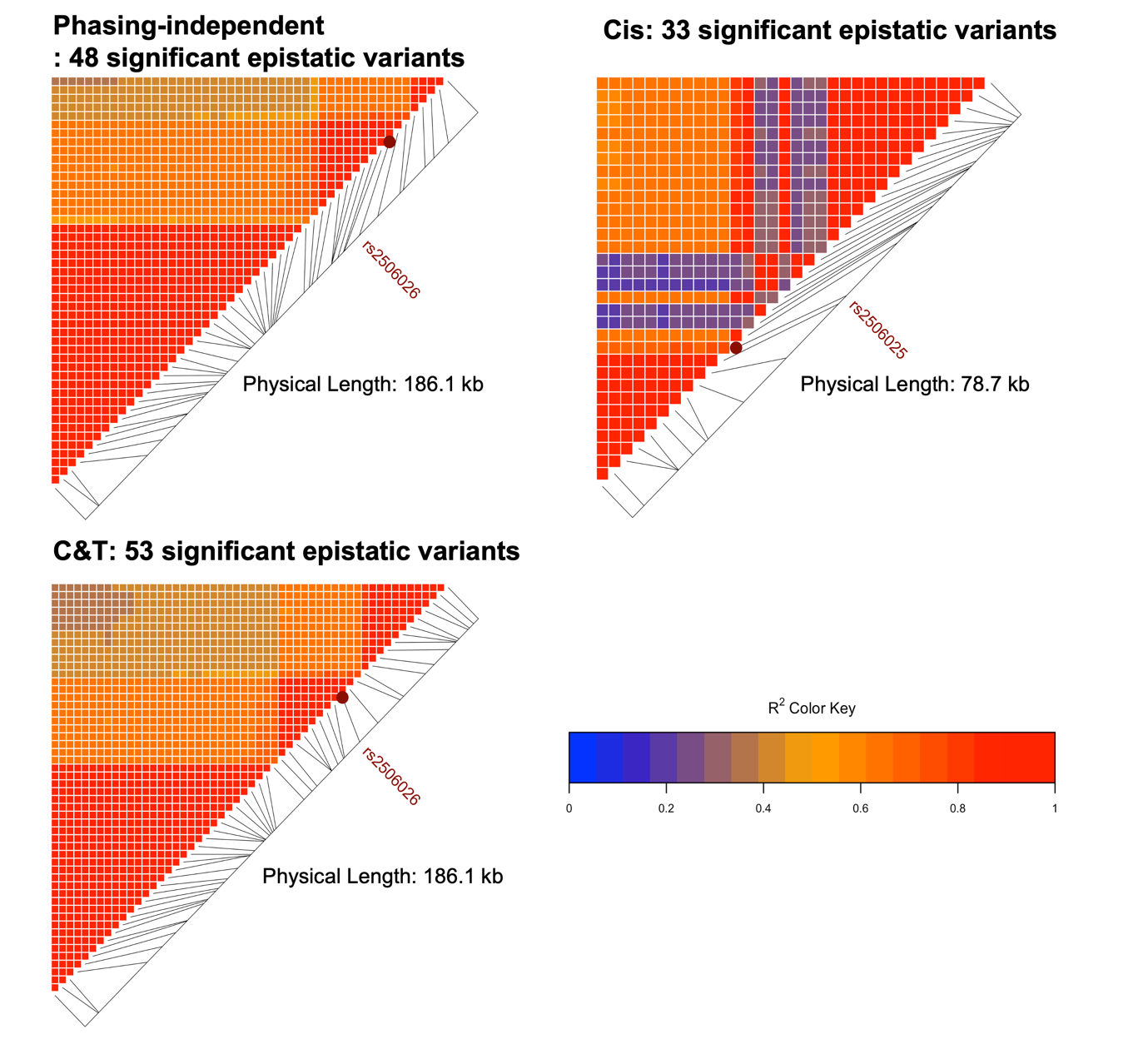
**

**Figure S2. The LD heatmap plots for the significant epistatic variants identified by phasing-independent, cis, and trans models**

The top epistatic variant for each model is highlighted by the rsID.

**
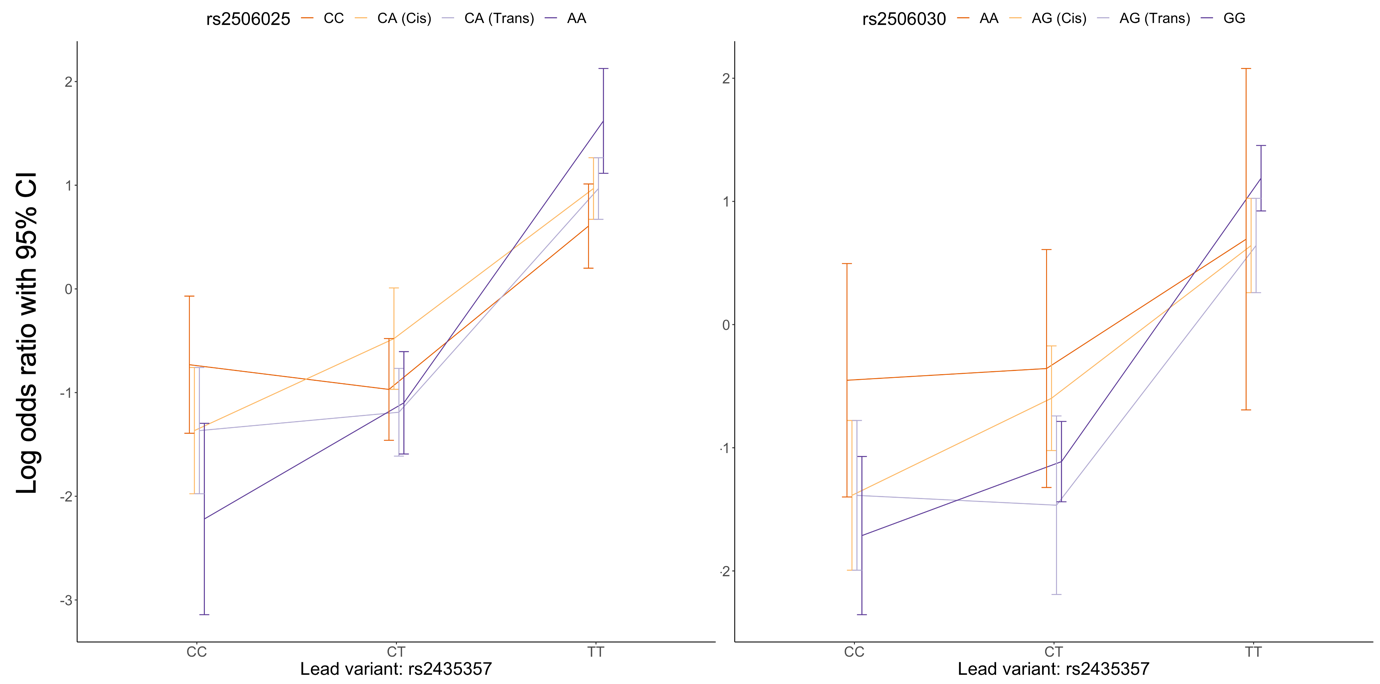
**

**Figure S3**. **The epistatic effects in log odds between the epistatic variants with the lead variant (rs2435357) in *RET***

On the left panel, CA (Cis) refers to the scenario where the risk allele (A) in rs2506025 and the risk allele (T) in rs2435357 are inherited from the same parent; CA (Trans) refers to the scenario where the risk allele (A) in rs2506025 and the risk allele (T) in rs2435357 are inherited from the different parents; Similarly, on the right panel AG (Cis) refers to the scenario where the risk allele (A) in rs2506030 and the risk allele (T) in rs2435357 are inherited from the same parent; AG (Trans) refers to the scenario where the risk allele (A) in rs2506030 and the risk allele (T) in rs2435357 are inherited from the different parents.

**
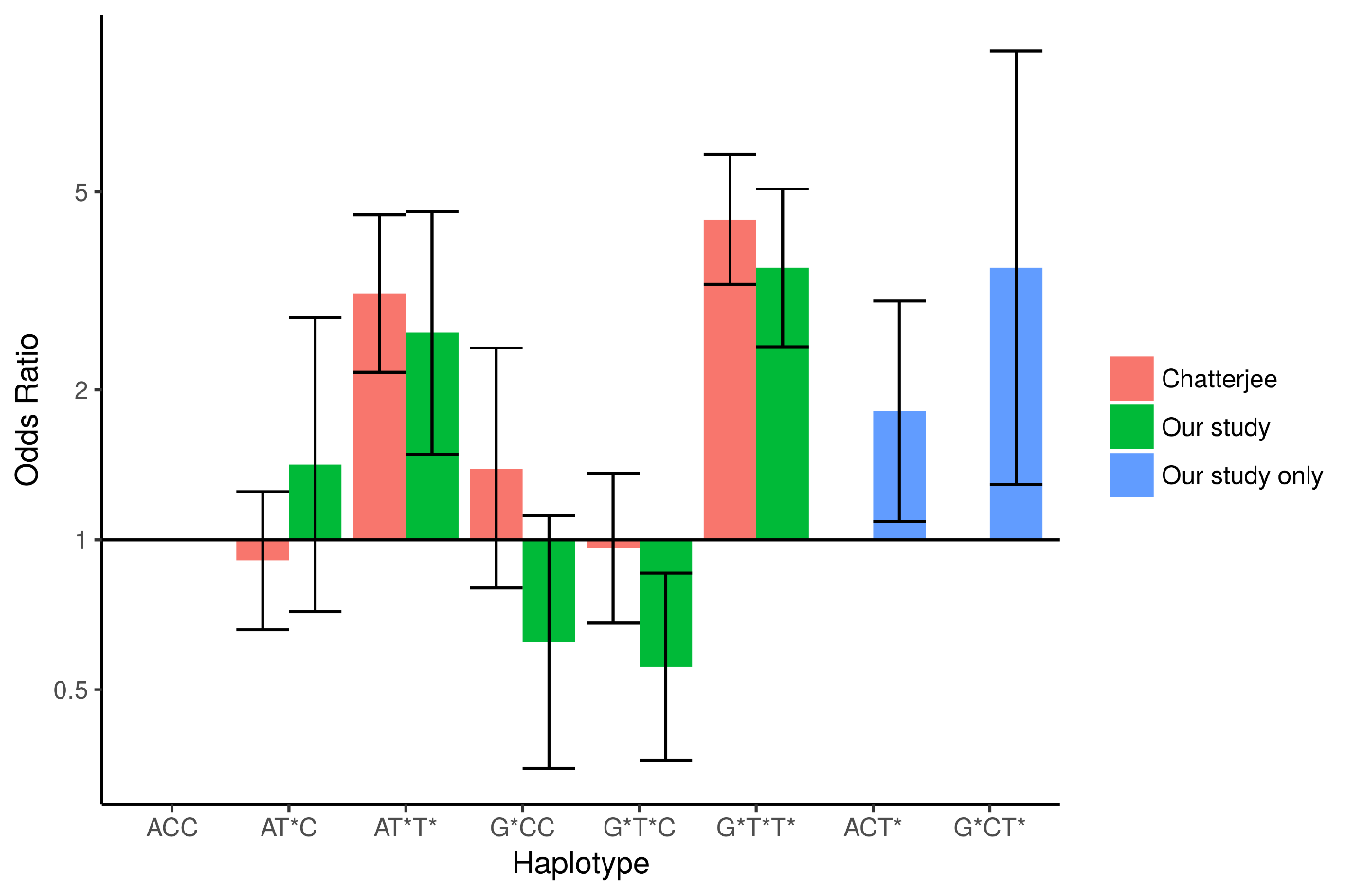
**

**Figure S4. The comparisons of odds ratios in eight haplotypes tagged by three SNPs** (rs2506030 (*RET-7*), rs7069590 (*RET-5.5*), rs2435357 (*RET+3*))

The odds ratios of eight haplotypes are compared to Chatterjee *et al.* (2016). The box plots covers the odds ratio estimates and 95% confidence intervals; ACC is the reference haplotype; “*” denoting the risk allele; Note that haplotypes ACT* and G*CT* were not examined in Chatterjee *et al.* (2016) because of small sample size.

**
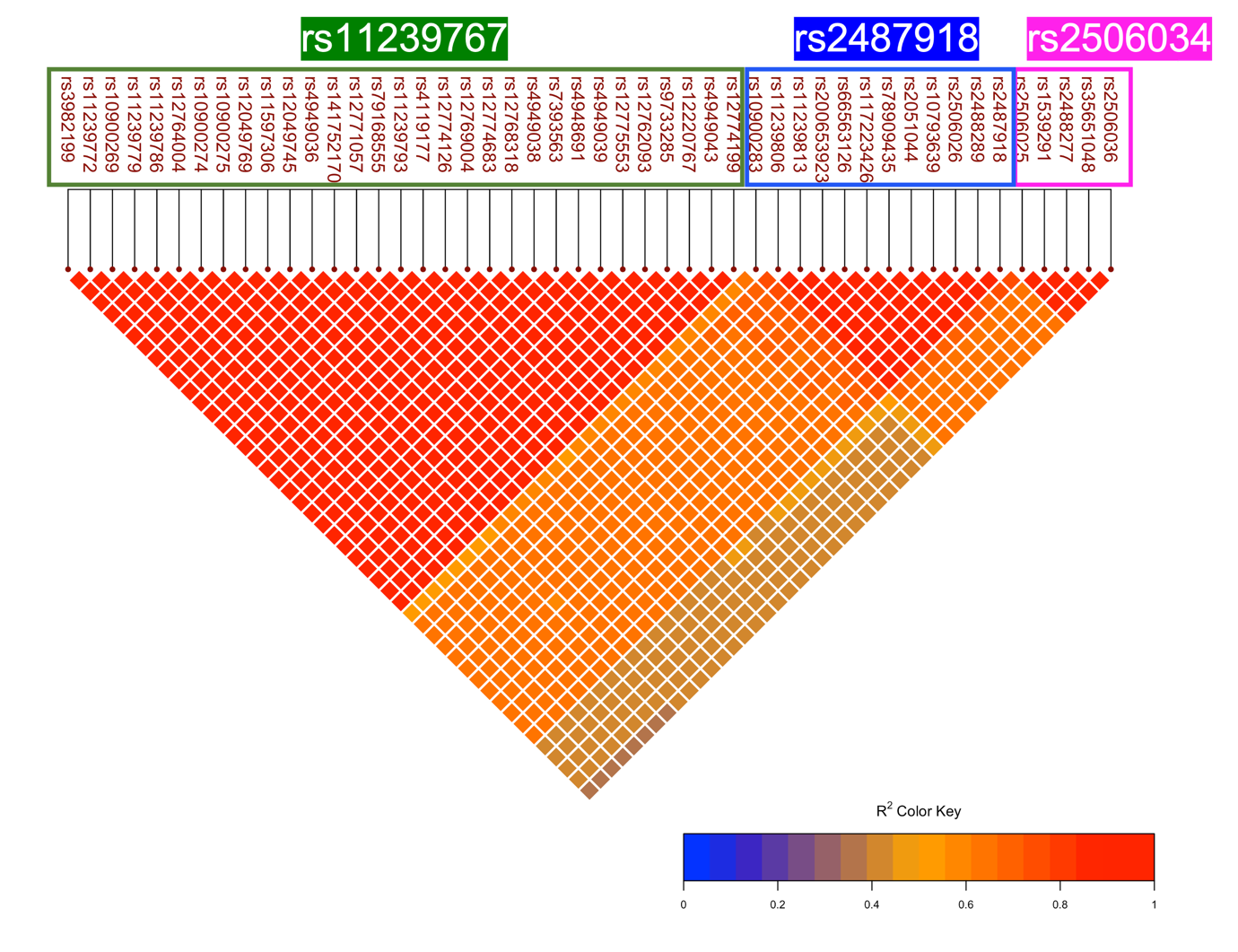
**

**Figure S5. Three proxy variants for the 48 identified epistatic variants**

The best proxy variants were selected to represent the groups of epistatic variants based on the underlying LDs. We used the reference data from Eastern Asian.

**
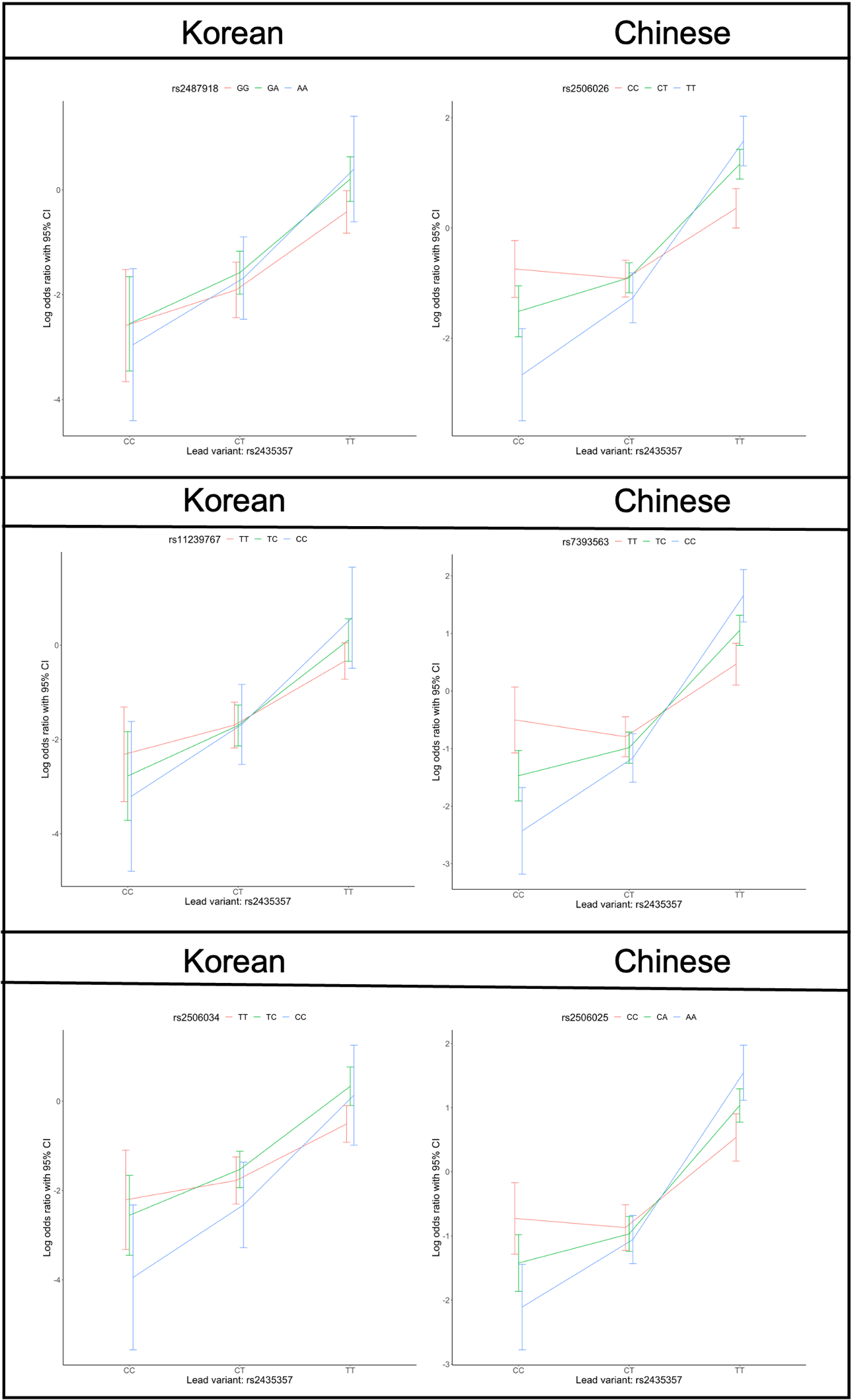
**

**Figure S6. Log odds comparison between epistasis variants in Korean and Chinese dataset.**

rs2505998 was used as the proxy var for the RET lead var (rs2435357) in the Korean dataset, where A allele in the proxy var rs2505998 corresponds to T allele in rs2435357, G allele in the proxy var rs2505998 corresponds to C allele inrs2435357.
